# Association between malformation type, location and functional deficits in lymphatic malformations of the head and neck in children

**DOI:** 10.1007/s00405-023-07844-x

**Published:** 2023-01-25

**Authors:** Susanne Wiegand, G. Wichmann, A. Dietz, J. A. Werner

**Affiliations:** 1 Department of Otolaryngology, Head and Neck Surgery, University of Leipzig, Liebigstr. 12, 04103 Leipzig, Germany; 2 University Hospital Essen, Essen, Germany

**Keywords:** Lymphatic malformation, Cologne disease score, Vascular malformation, Lymphangioma

## Abstract

**Purpose:**

Lymphatic malformations (LM) are congenital malformations of the lymphatic system, mainly located in the head and neck area. They can be staged based on location according to de Serres and based on different morbidity items using the Cologne Disease Score (CDS), a clinical staging system. In many cases, functional impairment greatly affects the life of patients suffering from lymphatic malformations. The present study aims to analyze a cohort of pediatric patients with LM.

**Methods:**

A retrospective analysis of 144 pediatric patients with head and neck LM was performed. Location, type of malformation (microcystic, macrocystic, mixed), scoring according to two different scoring systems and therapy were analyzed. Kruskal–Wallis test was used to analyze the difference in CDS between the patient groups and Dunn’s test was used for post-hoc pairwise comparison.

**Results:**

The average age at presentation was 6.1 years. The most common sites were neck (47%), cheek/parotid gland (26%), tongue (17%) and orbit (8%). Macrocystic malformations dominated the lateral neck, while microcystic malformations were predominantly localized in the tongue and floor of mouth. Macrocystic malformations (mean CDS 9.44) were associated with significantly better CDS than microcystic (mean CDS 7.11) and mixed (mean CDS 5.71) malformations (*p* < 0.001). LM in stage V according to de Serres had the lowest values (mean CDS: 4.26). The most common therapeutic procedures were conventional surgical (partial) resection, laser therapy and sclerotherapy with OK-432.

**Conclusions:**

There is an association between malformation type, location according to de Serres and CDS in children with LM of the head and neck. Patients with microcystic and mixed malformations in stage V had lowest CDS levels.

## Introduction

Lymphatic malformations are lesions of unknown aetiology usually diagnosed in infancy and childhood. They have equal sex incidence and present in various sites but most frequently occur in the head and neck region [1]. The cysts of lymphatic malformations are filled with proteinaceous lymph fluid [2]. Depending upon the location and surrounding tissues the cysts can vary in size. Therefore, lymphatic malformations can be characterized into macrocystic (cyst diameter > 1 cm), microcystic (cyst diameter < 1 cm), or mixed [3].

The way in which lymphatic malformations develop remains unknown, although several hypotheses regarding their origin have been postulated [1]. Acquired factors potentially contributing to their development include trauma, inflammation, and hormonal changes. Lymphatic malformations of the neck can be associated with syndromes (trisomy, Turner syndrome, Noonan syndrome, fetal alcohol syndrome), but most are sporadic. In many cases functional impairment and aesthetic deformity greatly affect the life of patients suffering from lymphatic malformations.

The first clinical staging system based on the patients’ symptoms was proposed by Wittekindt et al. in 2006 [4]. The Cologne Disease Score (CDS) is a clinical staging system for children with lymphatic malformations of the head and neck based on morbidity items disfigurement, dysphagia, dysphonia, and dyspnea. Moreover, an observer statement toward progression contributes to the CDS [4].

De Serres et al. [5] proposed in 1995 a classification system based on the location of lymphatic malformations. This classification divides lymphatic malformations according to their location into five stages. In stage I, the spread is unilateral infrahyoidal, stage II is unilateral suprahyoidal, stage III lesions are located unilateral supra- and infrahyoidal, stage IV lesions bilateral suprahyoidal, and stage V lesions bilateral supra- and infrahyoidal [5].

The aim of the present study was to analyze a cohort of children and adolescents with lymphatic malformations regarding these scores to find possible associations between malformation type, location and functional deficits.

## Patients and methods

A retrospective analysis of medical records of 144 children and adolescents (0–17 years) with lymphatic malformations of the head and neck was performed regarding gender, age at presentation, morphologic type, location, and treatment. The diagnosis of lymphatic malformation was established by clinical characteristics, magnetic resonance imaging and colour duplex ultrasound.

117/144 patients were scored by the classification of de Serres et al. which divides lymphatic malformations in five stages according to their location [5]. 122/144 patients were classified using the Cologne Disease Score (CDS) documentation sheet assessing five items (respiration, nutrition, speech, cosmetic appearance, progression) rated each with zero, one or two points [4]. A maximum score of ten points can be reached in case of five normal parameters. Kruskal–Wallis test was used to analyze the difference in CDS scores between the patient groups and Dunn’s test was used for post-hoc pairwise comparison.

## Results

**Fig. 1:**
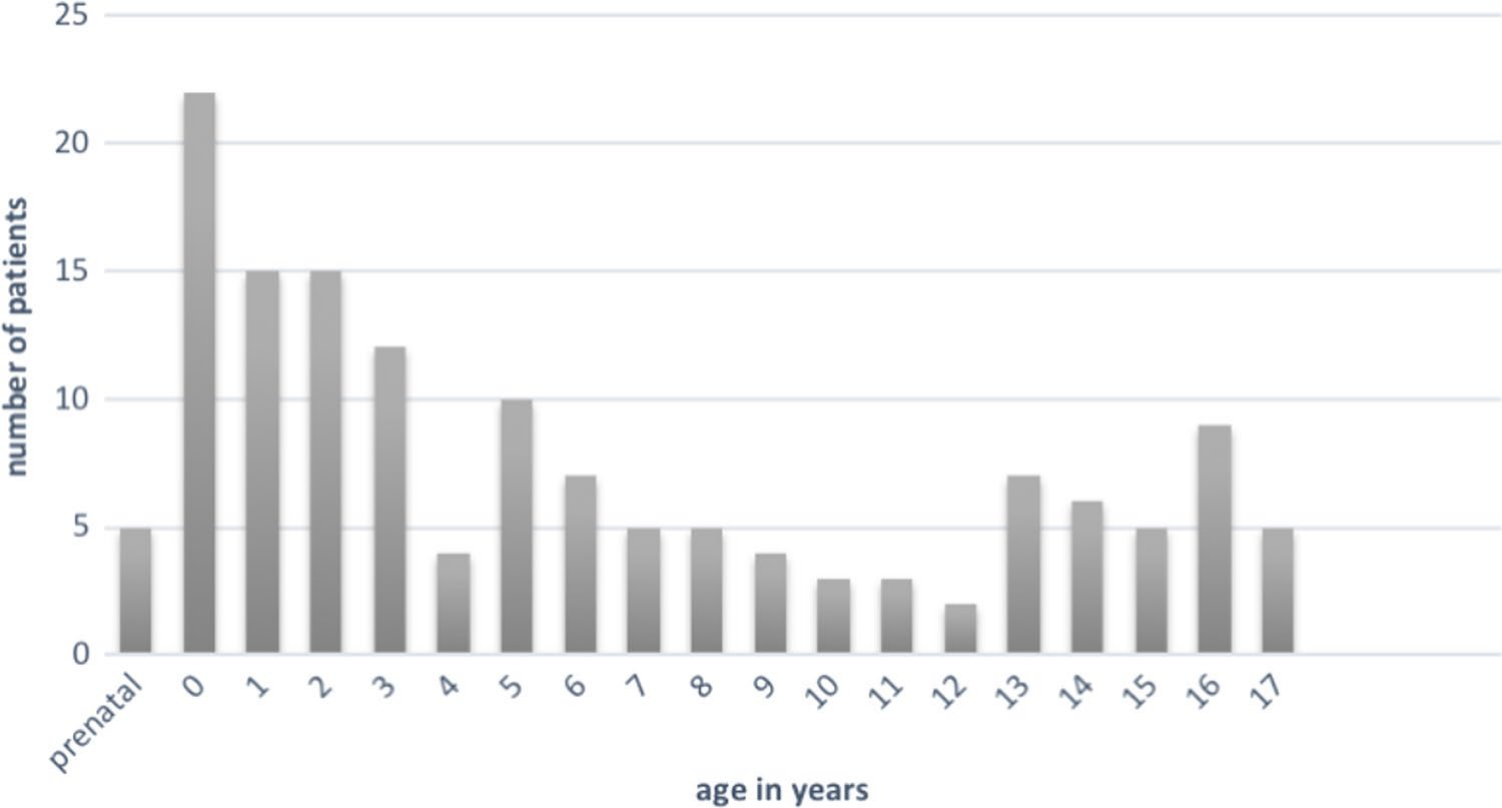
Age at presentation

In all, 144 (74 male/70 female) children and adolescents with lymphatic malformations of the head and neck were analyzed. The average age at first presentation was 6.1 years (Fig. 1). Four children were delivered by EXIT (ex utero intrapartum treatment) procedure due to prenatally diagnosed lymphatic malformations with the risk of fetal airway obstruction.

**Fig. 2:**
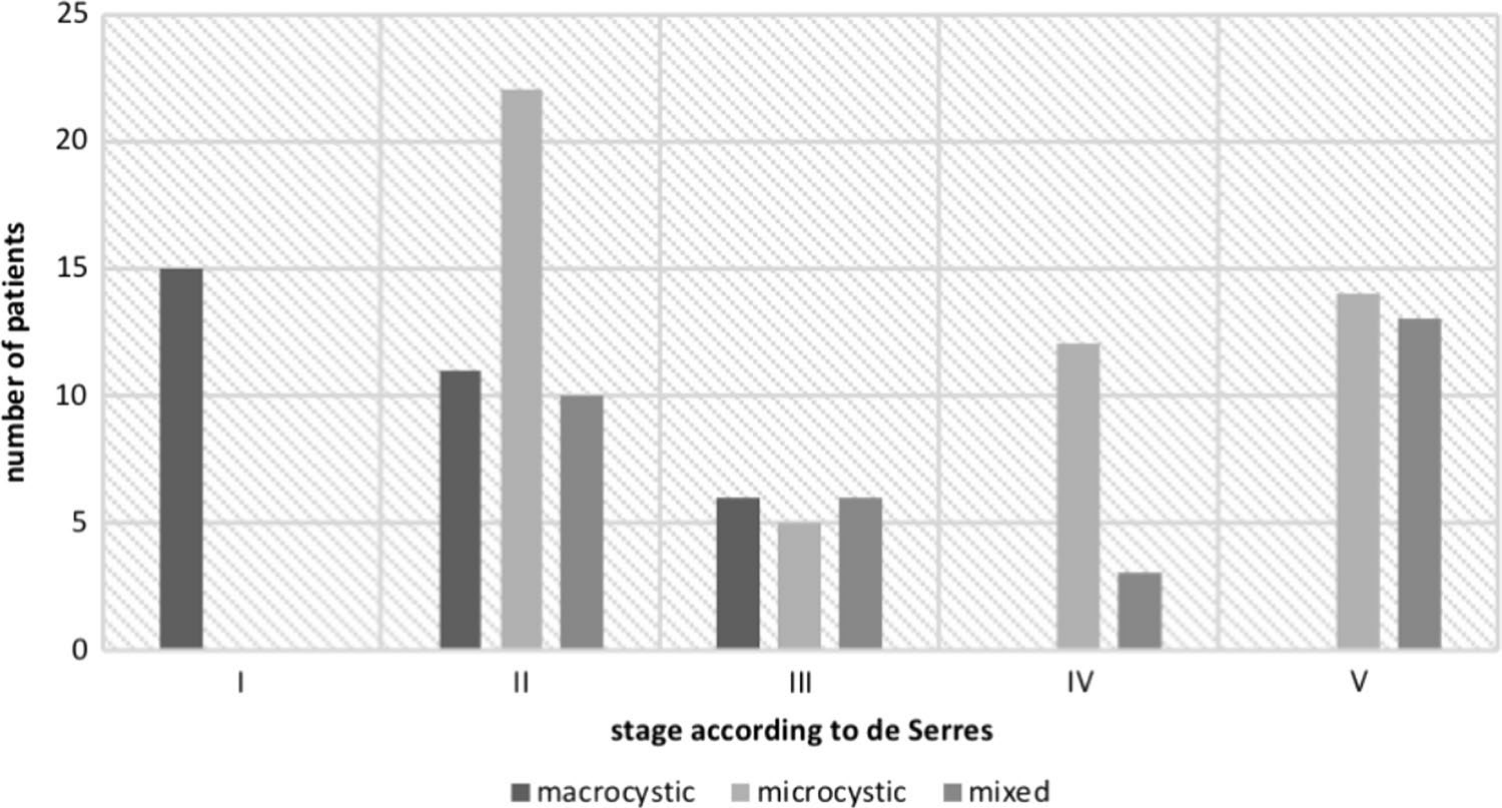
Distribution of macrocystic, microcystic and mixed lymphatic malformations according to the staging system of de Serres et al. [5]

The most common sites were cervical regions (47%), cheek/parotid gland (26%), tongue (17%) and orbit (8%). Macrocystic malformations were observed in 32 patients (22.2%), microcystic lesions in 55 patients (38.2%), and mixed lymphatic malformations in 35 patients (24.3%). In 15.3% of patients, the morphologic type of the lesion remained unknown due to missing documentation. In all, mixed lymphatic malformations were bigger in size than microcystic and macrocystic lymphatic malformations. According to classification of de Serres [1995], 117 patients could be evaluated. 15 patients (12.8%) were classified stage I, 43 patients (29.3%) stage II, 17 (14.5%) stage III, 15 (12.8%) stage IV, and 27 (23.1%) stage V (Fig. 2). Macrocystic malformations dominated in the lateral neck, while microcystic malformations were predominantly localized in the tongue and floor of mouth.

**Fig. 3:**
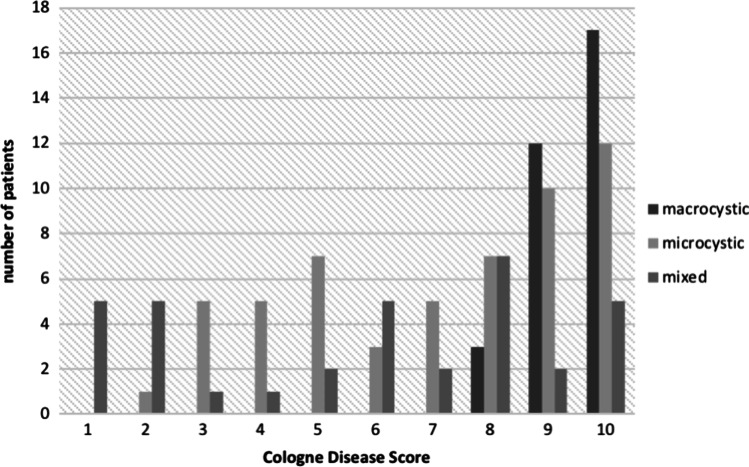
Distribution of the CDS of macrocystic, microcystic and mixed lymphatic malformations

122 patients could be evaluated regarding the CDS (Fig. 3). Macrocystic malformations (mean CDS 9.44) were associated with better CDS than microcystic (mean CDS 7.11) and mixed (mean CDS 5.71) malformations. All patients with macrocystic lymphatic malformations belonged to the moderate disease group (CDS: 8–10 points), whereas patients with advanced (CDS: 5–7 points) or severe disease (CDS: 0–4 points) had microcystic or mixed lymphatic malformations. The Kruskal–Wallis test indicated that there is a significant difference in the CDS scores depending on malformation type (*χ*^2^ = 32.74, *p* < 0.001). The post-hoc Dunn's test using a Bonferroni corrected alpha of 0.017 indicated that the mean ranks of the macrocystic and mixed malformations (*p* < 0.001) and macrocystic and microcystic malformations (*p* < 0.001) are significantly different, while the difference between microcystic and mixed lymphatic malformations is not significant (*p* = 0.057).

**Fig. 4:**
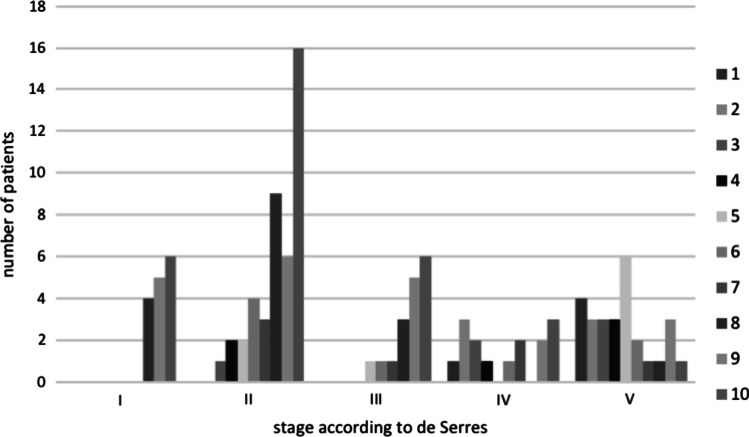
Distribution of the CDS according to the staging system of de Serres et al. [5]

**Fig. 5:**
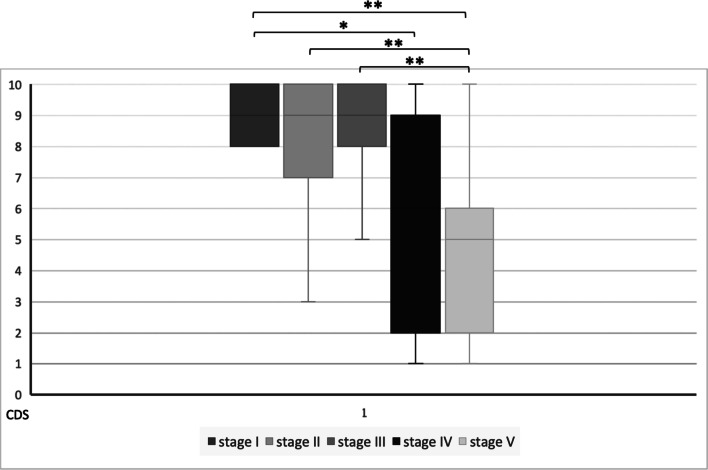
Box plot demonstrating CDS scores for the different stages according to de Serres [5]. Dunn’s test with a Bonferroni corrected alpha of 0.005 indicated significant differences between some of the groups (*p = 0.003, **p < 0.0001)

Malformations in stage V according to de Serres had the lowest values ​​evaluated by means of CDS (mean CDS: 4.26). Patients with lymphatic malformations stage I had a mean CDS of 9.13, while stage II patients had a mean CDS of 7.94, stage III patients of 8.65 and stage IV patients of 6.33 (Fig. 4). The Kruskal–Wallis test indicated that there is a difference in CDS scores between the different stages (*χ*^2^ = 37.07, *p* < 0.001). The box plot and post-hoc Dunn's test are shown in Fig. 5.

Nearly, one-third of the patients was previously treated at other hospitals before. The most common therapeutic procedures in our hospital were conventional surgical (partial) resection (47.0%), laser therapy (17.4%) and sclerotherapy with OK-432 (8.3%). The watch-and-wait policy was indicated in 27.7% of the patients. Microcystic and mixed malformations were less frequently treated but had worse outcome after treatment especially if compared to macrocystic malformations.

## Discussion

Lymphatic malformations affect one in every 2000–4000 live births [6]. Large lymphatic malformations can be diagnosed in utero using ultrasound as early as the beginning of the second trimester. The EX utero Intrapartum Treatment (EXIT) is a controlled technique to allow partial fetal delivery via modified Cesarean section with establishment of a safe fetal airway by intubation, bronchoscopy, or even tracheotomy, while fetal oxygenation is maintained through utero-placental circulation [7]. Four patients were delivered by EXIT procedure in this series.

Lymphatic malformations mainly develop in the cervicofacial region, generally below a normal-colored skin except when intralesional hemorrhage occurs [8]. Unfortunately, even small lymphatic malformations have the potential to become clinically significant, as acute infections and hormonal changes can lead to significant growth. Therefore, significant aesthetic and functional impairment can occur including compromise of the respiratory or digestive tract, speech impairment, dental malocclusion and vision loss. [6]

The goal of any treatment is to prevent functional deficits and to obtain a good aesthetic result. Complete resection of lymphatic malformations is the treatment of choice but often difficult because of their infiltrative nature and their intertwining with adjacent tissues. Due to diffuse infiltration in adjacent tissues especially in microcystic and mixed lesions a complete surgical excision often is impossible as the function has to be maintained. Whenever surgical intervention is impossible due to infiltrative growth into encompassing structures, sclerotherapy is the treatment of choice especially in macrocystic lesions [9]. Only in the absence of severe symptoms a conservative treatment strategy should be considered. Different systemic medications have been suggested to treat lymphatic malformations, but only limited evidence exists regarding their efficacy beyond small case series [10]. Sirolimus might be an effective treatment for patients with extensive lymphatic malformations and expands the range of therapeutic options; however, randomized controlled trials are lacking [11].

In the present study, the charts of children with lymphatic malformations of the head and neck were analyzed with regard to the items of the Cologne Disease Score. Patients with macrocystic malformations had significantly better CDS scores than patients with microcystic or mixed disease. All patients with macrocystic lymphatic malformations belonged to the moderate disease group (8–10 points) indicating that they have a lower morbidity, whereas patients with advanced (5–7 points) or severe disease (0–4 points) had microcystic or mixed lymphatic malformations. This distribution seems to be associated with the location of the disease. Macrocystic malformations were often located in the lateral neck, while malformations of the tongue and floor of mouth were typically microcystic.

Regarding the de Serres classification [5] for lymphatic malformations of the head and neck based on disease extent and anatomic location, we could demonstrate in the present study that there were patients in all stages. However, patients with lymphatic malformations stage IV and V had considerably lower CDS scores. This result affirms a major impact of the site of the lesion on symptoms and functional deficits. Other studies indicated that complication rate after surgery and the number of necessary surgical interventions correlated and increased with the stage number [5, 12–15].

Our study had limitations due to the heterogeneity of the LM patient characteristics and the fact that it is a retrospective study. There was variability in time elapsed, since lymphatic malformations were diagnosed. Inherent to the design we have to deal with missing data which could have impacted the presented results. Not every case in this series was scored with both systems. Only 122 of 144 patients were scored using the CDS, while 117 of 144 patients were classified according to de Serres [5]. Follow-up often was not documented as the hospital is a center for patients with vascular anomalies which often come from distant regions. Finally, given that the study is retrospective, it can only demonstrate association, not causation.

## Conclusion

There is an association between location according to de Serres, malformation type and functional deficits in children with lymphatic malformations of the head and neck. In the analyzed cohort, patients with microcystic and mixed malformations in stage V were associated with the most severity functional impairments and thus had the lowest values ​​in evaluation by means of CDS.

## Data Availability

All data analysed during this study are included in this published article.
